# Fibroblast growth factor 7 releasing particles enhance islet engraftment and improve metabolic control following islet transplantation in mice with diabetes

**DOI:** 10.1111/ajt.16488

**Published:** 2021-02-02

**Authors:** Salamah M. Alwahsh, Omar Qutachi, Philip J. Starkey Lewis, Andrew Bond, June Noble, Paul Burgoyne, Nik Morton, Rod Carter, Janet Mann, Sofia Ferreira‐Gonzalez, Marta Alvarez‐Paino, Stuart J. Forbes, Kevin M. Shakesheff, Shareen Forbes

**Affiliations:** ^1^ Centre for Regenerative Medicine University of Edinburgh Edinburgh UK; ^2^ School of Pharmacy University of Nottingham University Park Nottingham UK; ^3^ BHF Centre for Cardiovascular Science University of Edinburgh Queen’s Medical Research Institute Edinburgh UK; ^4^ Joint MD Program College of Medicine and Health Sciences Palestine Polytechnic University Hebron Palestine

**Keywords:** animal models: murine, diabetes: type 1, islet transplantation, regenerative medicine, translational research/science

## Abstract

Transplantation of islets in type 1 diabetes (T1D) is limited by poor islet engraftment into the liver, with two to three donor pancreases required per recipient. We aimed to condition the liver to enhance islet engraftment to improve long‐term graft function. Diabetic mice received a non‐curative islet transplant (*n* = 400 islets) via the hepatic portal vein (HPV) with fibroblast growth factor 7‐loaded galactosylated poly(DL‐lactide‐co‐glycolic acid) (FGF7‐GAL‐PLGA) particles; 26‐µm diameter particles specifically targeted the liver, promoting hepatocyte proliferation in short‐term experiments: in mice receiving 0.1‐mg FGF7‐GAL‐PLGA particles (60‐ng FGF7) vs vehicle, cell proliferation was induced specifically in the liver with greater efficacy and specificity than subcutaneous FGF7 (1.25 mg/kg ×2 doses; ~75‐µg FGF7). Numbers of engrafted islets and vascularization were greater in liver sections of mice receiving islets and FGF7‐GAL‐PLGA particles vs mice receiving islets alone, 72 h posttransplant. More mice (six of eight) that received islets and FGF7‐GAL‐PLGA particles normalized blood glucose concentrations by 30‐days posttransplant, versus zero of eight mice receiving islets alone with no evidence of increased proliferation of cells within the liver at this stage and normal liver function tests. This work shows that liver‐targeted FGF7‐GAL‐PLGA particles achieve selective FGF7 delivery to the liver‐promoting islet engraftment to help normalize blood glucose levels with a good safety profile.

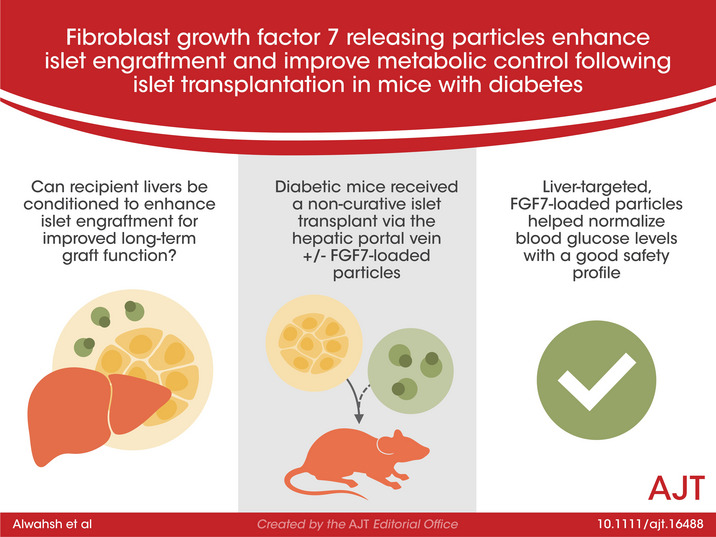

## Graphical abstract

Cotransplantation of islets with liver‐targeted, biodegradable microparticles loaded with fibroblast growth factor 7 into livers of diabetic mice leads to short‐term hepatocyte proliferation and improves islet engraftment with superior long term glycemic control versus transplantation with islets alone. See the editorial on page 2927.

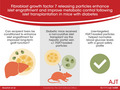

**Abbreviations**
ASGPR: asialoglycoprotein receptor
BrdU: 5‐bromo‐2'‐deoxyuridine
FCS: fetal calf serum
FGF7: fibroblast growth factor 7
FGF7‐GAL‐PLGA: FGF7‐loaded galactosylated poly(DL‐lactide‐co‐glycolic acid)
GFs: growth factors
H&E: hematoxylin and eosin
HGF: hepatic growth factor
HNF4α: hepatocyte nuclear factor 4α
HPV: hepatic portal vein
HSA: human serum albumin
IAH: impaired awareness of hypoglycemia
PSR: picrosirius red
SH: severe hypoglycemia
STZ: streptozotocin
T1D: type 1 diabetes
T3: triiodothyronine
VEGF: vascular endothelial growth factor

## 1 INTRODUCTION

Islets are clusters of polyhormonal cells including insulin‐secreting β‐cells. In type 1 diabetes (T1D), destruction of pancreatic β‐cells by autoimmune processes leads to insulin deficiency requiring insulin replacement. Severe hypoglycemia (SH) is a side effect of exogenous insulin and affects over 10% of those with T1D.^1^ Human islet allotransplantation stabilizes glycemic control and decreases the frequency of recurrent SH in T1D.^1^, ^2^, ^3^, ^4^, ^5^, ^6^, ^7^, ^8^ However, >60% of transplanted islets fail to engraft into the liver,^9^, ^10^ and islets from two to three pancreas donors per recipient are needed to impact glycemic control.^11^ Islets are avascular and following islet transplantation, the blood vessel supply between islets and the liver starts to form by day 3. The majority of islet loss occurs within the first 3 days posttransplant.^12^ Hypoxia due to a lack of a blood supply and inflammation contributes to islet loss.^13^ We hypothesized that a hepatic microenvironment favoring islet retention and vascularization in the early stages posttransplant would ameliorate early islet loss and aid long‐term function.

Preconditioning the host liver with growth factors (GFs) creates a receptive “niche” involving the re‐modeling and proliferation of liver cells.^14^, ^15^ Systemic GFs, such as triiodothyronine (T3),^16^, ^17^ hepatocyte growth factor (HGF),^14^ and fibroblast growth factor 7 (FGF7),^18^ have been used to increase rat liver cell proliferation and enhance the efficiency of retroviral gene delivery. FGF7 is a small polypeptide member of the FGF family that binds to the FGF7 receptor and has proliferative and antiapoptotic effects on epithelial cells including hepatocytes.^18^, ^19^

GFs may promote islet engraftment through: (1) liver cell proliferation and immediate islet “trapping”; (2) upregulation of VEGF, promoting vasculogenesis and early islet vascularization;^20^, ^21^ (3) anti‐inflammatory activity, thereby aiding islet survival;^22^, ^23^ and (4) inhibition of T cell–mediated immune effects^24^ reducing islet rejection.

Administering GFs systemically is limited by their short half‐life, low tissue penetration, and effects on multiple organs. Low GF concentrations in the targeted organ necessitate dose escalation with off‐target effects.^25^ We tested targeted GF delivery to the liver to promote short‐term liver cell proliferation, enhance islet engraftment, and improved metabolic control in a mouse model of T1D.

PLGA polymer is a biodegradable material used in medical devices. The polymer matrix achieves desirable release kinetics based on the polymer hydration profile.^26^ For targeted delivery to the liver, the PLGA polymer may have galactose added to it,^27^, ^28^ exploiting asialoglycoprotein receptor (ASGPR)‐mediated endocytosis. There are ~25 000 ASGPR in the hepatocyte plasma membrane with a specific binding affinity toward the galactose moiety attached on the PLGA particles.^28^ Based upon its ability to induce proliferation in the liver following systemic use, a GF was selected for use in an engineered polymer for targeted delivery to the liver.

Our aim was then to create a microenvironment in the liver suitable for early islet engraftment, using the GF‐PLGA polymer‐associated complex. In order to do this we: (1) characterized the biodistribution and release kinetics of several formulations and particle sizes in vivo; (2) identified an optimal particle size and dose; (3) co‐transplanted GF‐loaded galactosylated PLGA (GAL‐PLGA) particles concurrently with a non‐curative mass of islets via the clinically relevant hepatic portal vein (HPV) into diabetic mice and monitored glycemic control over a 6‐week period with histological assessments of islet engraftment in the liver.

## 2 MATERIALS AND METHODS

### 2.1 Animals

Male C57Bl/6 mice (8–10 weeks old, Harlan Laboratories) were housed under standard conditions in a 14‐h light to 10‐h dark cycle and given standard chow and water ad libitum.

### 2.2 Injection of growth factors and proliferation of cells within liver

In short‐term experiments, 12‐week‐old C57Bl/6 mice (*n* = 8/group) received the following GFs or vehicle: Group (1) recombinant FGF7 1.25 mg/kg subcutaneously (s.c.) (Sobi Pharmaceuticals), Group (2) HGF 250 μg/kg i.v. (R&D Systems^™^), Group (3) T3 4 mg/kg s.c. (R&D Systems^™^), Group (4) all three GFs, and Group (5) 100‐μl saline (vehicle), at day −2 and day 0. These doses were based on previous studies^18^ with FGF7, which demonstrated that at 24 h following one injection of FGF7 at high dose (5 mg/kg), three of eight mice were anorectic and hypoglycemic (blood glucose levels between 3.0 and 3.9 mmol/L) with signs of distress (Table S1). Therefore, lower doses of FGF7 were used with no adverse effects. Mice were pulsed with BrdU (1 mg dissolved in PBS) via intraperitoneal (i.p.) injection 48 h later and culled 1 h afterwards. Liver lobes were processed for immunohistochemistry (see below) with the antibodies listed in Table S2. The GF associated with the greatest liver cell proliferation was selected and given s.c. prior to administering islets via the HPV, to determine if glycemic control was improved. This GF was subsequently incorporated into galactosylated PLGA particles.

### 2.3 FGF7 and effects on insulin secretion and oxygen consumption rates of islets in vitro

Islets (*n* = 20 islets per well in triplicate) were incubated in 0 (control), 5 ng/ml, and 30 ng/ml FGF7 for 24 h. FGF7 concentrations were based on concentrations released from 1‐mg FGF7‐GAL‐PLGA particles. Glucose‐stimulated insulin secretion (GSIS) was measured with insulin quantified by ELISA (Mercodia)^10^ and oxygen consumption rates (OCRs) of islets were measured in triplicate as previously described.^10^

### 2.4 PLGA particle preparation, assembly, and characterization

PLGA 50:50 lactide:glycolide ratio (52 kDa, DL‐lactide, Lakeshore Biomaterials) was functionalized with lactobionic acid (LB, Sigma Aldrich) and fabricated from 5.5% PLGA in dichloromethane (DCM, Fisher) by a double emulsion method.^25^ The polymer solution, containing 0.1% w/v FGF7 and 0.9% human serum albumin (HSA, Sigma Aldrich) was homogenized stirred, filtered, and freeze dried. Particle size distribution (Coulter LS230, Beckman) was measured. Protein release (HSA+FGF7) was measured by bicinchoninic acid assay (Sigma Aldrich) for 21 days.

### 2.5 Biodistribution of particles via the HPV or tail vein

PLGA particles were rhodamine‐labeled. Mice (*n* = 3/group) received an injection of 1‐mg PLGA particles in 100‐µl 30% fetal calf serum (FCS). Non‐galactosylated PLGA particles (2, 10, and 22 µm mean diameter) and GAL‐PLGA particles (2, 10, and 26 µm mean diameter) were injected with a 30G needle via the HPV and via the tail vein (i.v.); vehicle injections were also run as controls. Mice were culled 24 h postinjection, blood samples collected by cardiac puncture, and the liver, lung, kidney, heart, and spleen were harvested for analysis.

### 2.6 Safety and efficacy studies with FGF7‐GAL‐PLGA particles

Safety and efficacy studies were performed with FGF7‐GAL‐PLGA particles (26 µm) in increasing doses. Twelve‐week‐old C57Bl/6 mice (six groups; *n* = 3–4/group) received an HPV injection of FGF7‐GAL‐PLGA particles (0.01, 0.1, 1, or 5 mg in 100‐μl 30% FCS), 30% FCS, or FGF7 s.c. (1.25 mg/kg; 100 µL, once daily for 2 days). Terminal serum samples were collected for LFTs, FGF7, and VEGF‐A, 72 h post first injection; livers were processed for immunohistochemistry (H+E staining). Liver homogenates were further analyzed for VEGF‐A concentrations.

Based on the data above, the effect of 26‐µm FGF7‐GAL‐PLGA particles (0.1 mg) on liver cell proliferation 72 h posttransplant was determined. C57Bl/6 mice (12‐week‐old; four groups; *n* = 3–4 per group) were injected via the HPV with: (1) FGF7‐GAL‐PLGA particles (0.1 mg), (2) GAL‐PLGA particles (0.1 mg), (3) 30% FCS ×2 (100 µl), and (4) FGF7 s.c. (1.25 mg/kg once daily for 2 days). Mice were pulsed with BrdU 1 mg i.p 1 h pre‐cull and livers were processed for immunohistochemistry (cell proliferation) as described.

### 2.7 Mouse islet isolation

Pancreatic islets were isolated from 12‐week‐old male C57Bl/6 mice by a collagenase digestion method,^10^ and islet purity was ≥90%.

### 2.8 Induction of diabetes in mice

C57Bl/6 mice (*n* = 8–10/group) received streptozotocin (STZ) (Sigma‐Aldrich) at 16–17 weeks old by administration of 180 mg/kg i.p. and classed as hyperglycemic if non‐fasted glucose levels were ≥17.0 mmol/L (306 mg/dl) for two consecutive days. Islet transplantations took place within 10 days of STZ.

### 2.9 Transplantation of islets with subcutaneous FGF7 and FGF7‐GAL‐PLGA particles

Diabetic C57Bl/6 mice (*n* = 6–8/group) were transplanted with: (1) 400 islets (in 200‐µl RPMI 1640 medium), and (2) 400 islets plus FGF7 1.25 mg/kg s.c. ×2 doses (48 h pretransplant and at time of transplant). Control experiments included non‐diabetic mice (*n* = 4/group) given (3) FGF7 1.25 mg/kg s.c. (100 µl) ×2 doses and (4) vehicle (saline [100 µl] s.c. ×2 doses). Body weight and venous blood glucose (OneTouch Verio, LifeScan) were monitored daily and mice were sacrificed 6 weeks posttransplant.

Further experiments included diabetic C57Bl/6 mice (*n* = 8–10/group) transplanted with: (1) 400 islets, (2) 400 islets plus 0.1‐mg FGF7‐GAL‐PLGA particles via the HPV, and (3) vehicle‐injected mice. Mice were monitored and at 6 weeks posttransplant, mice fasted overnight were given 1‐mg BrdU i.p. and administered a 2 g/kg IPGTT with glucose measurements at 15, 30, 60, 90, and 120 min afterwards and a plasma insulin at 60 min. Mice were subsequently culled and livers (*n* = 8–10/group) were analyzed for cell proliferation, vascularization, VEGF‐A, and fibrosis. Pancreases (*n* = 4/group) were analyzed for β‐cell proliferation. The insulin content of the remaining pancreases was extracted and analyzed.

In short‐term experiments, mice (*n* = 3/group) were transplanted with 0.1‐mg FGF7‐GAL‐PLGA particles plus 400 islets versus a group transplanted with 400 islets alone (*n* = 3/group) and sacrificed 72 h posttransplant. Livers were analyzed using immunohistochemistry for islet numbers (insulin and glucagon) and vascularization (CD31 and VEGF‐A).

### 2.10 Biochemical analysis

ALT, albumin, and bilirubin (Alpha Laboratories Ltd.) were analyzed on the Cobas Fara centrifugal analyzer (Roche), human FGF7 and insulin concentrations by ELISA (Thermo Scientific; Mercodia, respectively); insulin content of the pancreas was measured following weighing, homogenization, and sonication.^29^ VEGF‐A was quantified by ELISA in serum samples and in liver homogenates at 72 h and 6 weeks posttransplant (U‐PLEX Mouse VEGF‐A Assay, MSD).

### 2.11 Histological analysis

We quantified liver cell proliferation, islet engraftment, liver fibrosis, necrosis, and vascularization. Tissue was either fixed, embedded in paraffin, and cut serially (5 µm) or processed using cryosections (8–30 µm). In brief to analyze: (i) proliferation‐liver sections were immunostained with BrdU and hepatocyte nuclear factor 4α (HNF4α) to hepatocyte proliferation and total hepatocyte population, respectively, and (ii) PLGA particle detection‐rhodamine‐labeled particles were detected using fluorescence microscopy and quantified from an average of 11 × 40 fields per organ. To detect rhodamine‐labeled PLGA particles in Kupffer cells, the Kupffer cells were detected using a rat anti‐F4/80 immunostaining followed by an anti‐rat Alexa Fluor 488 secondary antibody. (iii) Islet engraftment β‐cells were quantified in ≥8 formalin‐fixed paraffin‐embedded (FFPE) sections of liver from all lobes (>50 µm apart [in Z orientation]). More than 15 non‐overlapping fields per section were evaluated, using ×20 magnification (Nikon Eclipse E600 fluorescent microscope). The average number of β‐cells detected per FFPE section was standardized to the total analyzed fields.^30^ (iv) Necrosis and fibrosis‐H&E stains were produced using a Shandon Varistain Automated Slide Stainer. Collagen fibers in the liver tissues were detected with picrosirius red (PSR) staining.^31^ (v) Vascularization was determined in paraffin‐embedded liver sections using immunofluorescence for CD31^32^ and the erythroblast transformation‐specific‐related gene (ERG): a transcription factor specific for endothelial cells;^33^ VEGF‐A quantification was attempted in liver sections using an immunofluorescence method.^10^, ^33^ For each immunostain, control procedures included isotype‐matched rabbit monoclonal antibodies. DAPI staining was performed to label nuclei. Slides were mounted using an aqueous medium and imaged using an Operetta High‐Content System (PerkinElmer).

### 2.12 Statistical analysis

Results are expressed as mean ± SEM unless otherwise stated. Significance was determined by unpaired *t* tests or one‐way ANOVA with Tukey's post hoc testing using Prism 6.0 software (GraphPad Software). A *p* < .05 was considered significant.

## 3 RESULTS

### 3.1 Subcutaneous FGF7 enhanced liver cell proliferation more than other GFs in short‐term experiments

**FIGURE 1:**
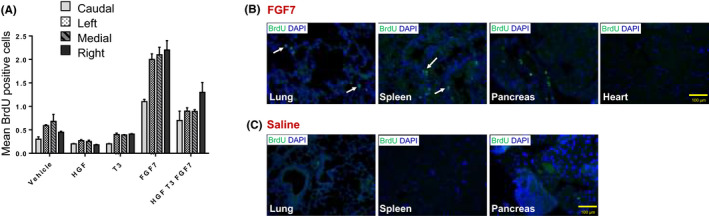
Cell proliferation in liver and other organs after injection of GFs. Mice received ×2 injections of the following GFs or vehicle 48 h apart (day 0 at transplant and day 2) and culled 48 h following the last injection of growth factor or vehicle. One hour before cull, BrdU was injected i.p. to detect cell proliferation. The number of BrdU‐positive cells (=proliferating cells) was evaluated by the Operetta system and Columbus software. (A) Vehicle (100 μl saline) s.c., HGF 250 μg/kg i.v., T3 4 mg/kg s.c., FGF7 1.25 mg/kg s.c, or combination of all three GFs. (B) Immunofluorescence staining for BrdU in various organs in mice receiving FGF7 1.25 mg/kg s.c. × 2 doses or (C) vehicle 100 μl saline s.c × 2 doses. Arrows indicate BrdU‐positive cells. DAPI (blue) indicate cell nuclei. Data represent the mean ± SEM

FGF7 1.25 mg/kg s.c. was associated with the greatest total proliferation of liver cells (parenchymal and non‐parenchymal) versus HGF 250 µg/kg i.v., T3 4 mg/kg s.c., and all three GFs in combination; all mice were given two injections of GFs, the first at the time of transplant and the second 48 h later with liver cell proliferation assessed 48 h following this (Figure 1A). Mice receiving FGF7 s.c. exhibited pronounced cell proliferation in all organs including the lungs, pancreas, heart, and spleen, as demonstrated by BrdU immunofluorescence staining versus controls (Figure 1B,C). Therefore, FGF7 was selected for further studies.

### 3.2 Subcutaneous FGF7 with islets did not control blood glucose levels more effectively than transplantation of islets alone

Mice with diabetes transplanted with 400 islets plus FGF7 1.25 mg/kg s.c (×2 injections) did not demonstrate improved glycemic control compared with mice transplanted with islets alone by 6 weeks, with no mice cured from their diabetes: glucose concentrations at 6 weeks: (mean ± SEM): 18.1 ± 2.2 vs. 19.2 ± 1.8 mmol/L, respectively (*p* = .80). Control normoglycemic mice receiving FGF7 (1.25 mg s.c. × 2 doses) versus vehicle‐treated mice showed no difference in glucose concentrations over a 6‐week period (mean ± SEM): 8.6 ± 0.4 vs. 8.3 ± 0.6 mmol/L, respectively (*p* = .91).

### 3.3 FGF7 has no effect on insulin secretion or OCR in short‐term in vitro studies with islets

FGF7 at a dose of 5 or 30 ng/ml had no effect on insulin secretion or OCR (Figure S1).

### 3.4 FGF7‐GAL‐PLGA particles released FGF7 predominantly in the first 48 h over a 21‐day period

**FIGURE 2:**
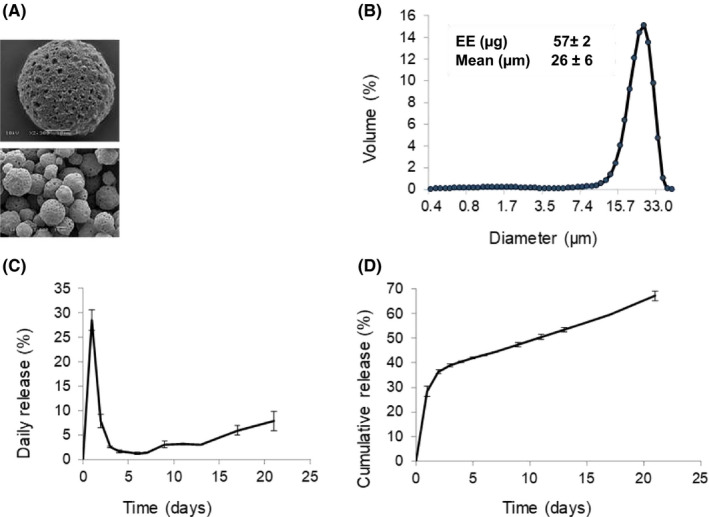
Characterization of GAL‐PLGA particles. (A) Representative scanning electron micrographs of particles. (B) Size distribution of particles with average size diameter and entrapment efficiency (%) for total protein. (C) Individual daily release percentage, and (D) cumulative release kinetics for the total protein payload over a 3‐week period. Data represent mean ± SD. EE, entrapment efficiency

Fabricated GAL‐PLGA particles were regular and spherical with porous surfaces (Figure 2A). The average diameter of the galactosylated particle was (mean ± SD) 26 ± 6 µm with 57 ± 2% FGF7 loading efficiency (Figure 2B).

The release kinetics showed an initial burst release phase, releasing ~one‐third of the FGF7 payload on day 1, declining to 8% release on day 2, and 3% on day 3. Release was maintained at 1% between days 4 and 6 increasing to ~8% release from days 9 to 21 (Figure 2C). Cumulative in vitro release profiles of the particles and FGF7 delivery dose (ng per mg particles) are shown over 3 weeks (Figure 2D; Table 1). For 0.1‐mg PLGA particles, the FGF7 content was 60 ng and a 70% release of FGF7 over 21 days was ~40 ng FGF7.

**TABLE 1:** Release of FGF7 (% and ng/1 mg particle) at the selected time points up to day 21

Day	Release (%)	Release in ng per 1 mg particles
1	28.4 (±2.1)	163.3 (±5.5)
2	7.8 (±1.3)	45.4 (±9.5)
3	2.5 (±1.5)	14.5 (±1.5)
4	1.6 (±0.2)	9.3 (±2.0)
5	1.3 (±0.07)	7.9 (±0.7)
6	1.1 (±0.2)	6.8 (±1.4)
7	1.3 (±0.1)	7.7 (±0.9)
9	3.0 (±0.7)	17.5 (±4.9)
11	3.1 (±0.1)	18.0 (±1.7)
13	2.9 (±0.06)	16.9 (±0.7)
17	5.9 (±0.9)	33.9 (±4.0)
21	7.2 (±2.0)	44.7 (±9.7)

Microparticles (FGF‐GAL‐PLGA – 25 mg) were set up in triplicate and suspended in 1‐ml PBS, gently rocked on a three‐dimensional shaker (Gyrotwister, Fisher Scientific UK Ltd) at 5 rpm in a humidified incubator at 37°C and supernatant collected at specified time points (mean ± SEM).

### 3.5 GAL‐PLGA particles administered via HPV injection specifically target the liver

**FIGURE 3:**
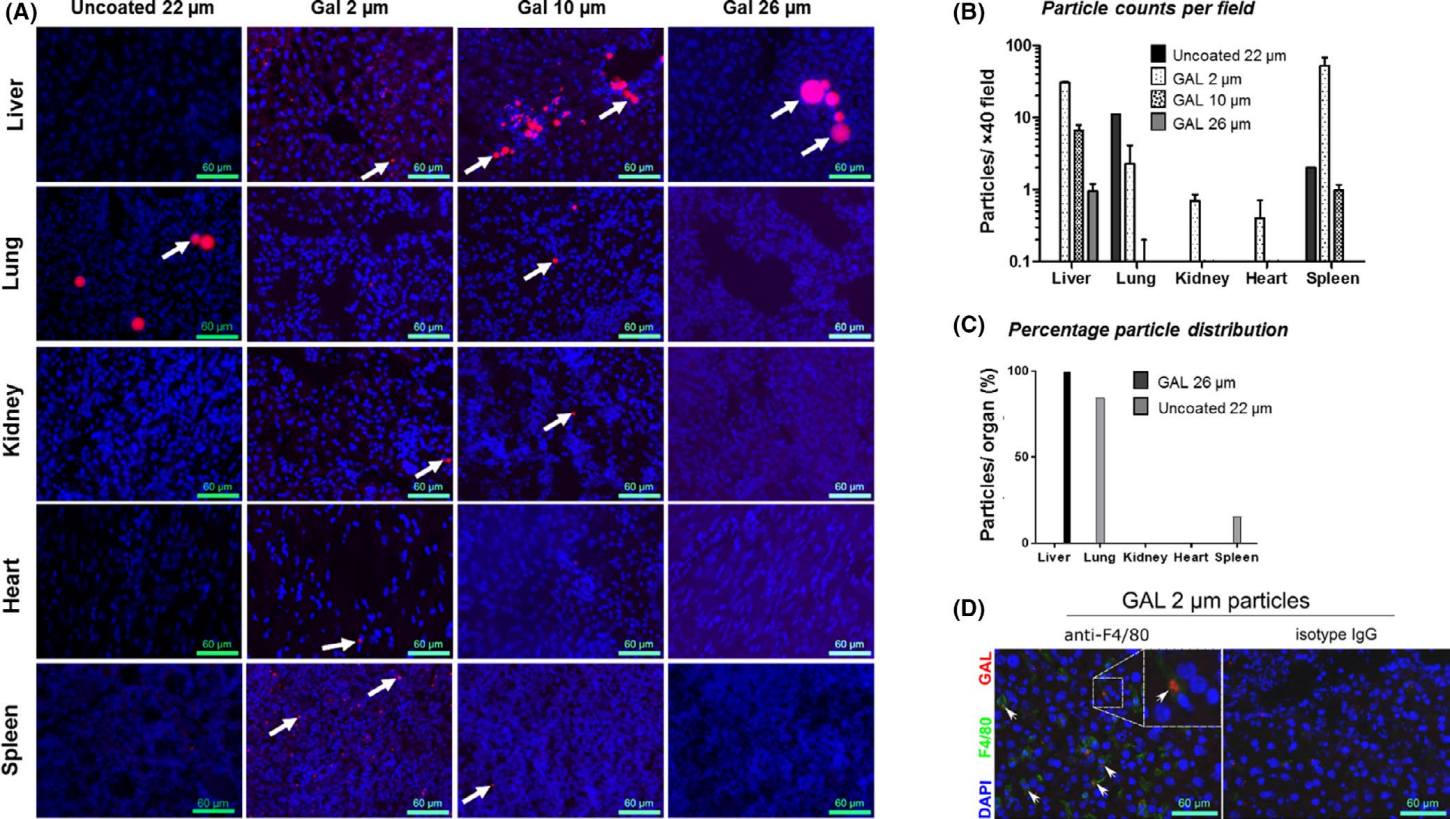
Hepatic portal vein injection of 26‐μm GAL‐PLGA particles via the HPV provides specific hepatic localization. PLGA particles non‐galactosylated (22 µm) and GAL‐PLGA (2, 10, and 26 µm) were injected into mice (1 mg, HPV) to determine particle distribution in organs. (A) Representative fluorescent images (×40) of organs extracted 24 h after injection of particle formulations. Cryosections (30 µm) were fixed and stained: DAPI (blue, cell nuclei); fluorescent particles (red epifluorescence) are highlighted (white arrows). (B) Mean particle counts (logarithm, from 11 slides) quantified per tissue grouped in formulations indicated. Bars represent the mean ± SD, *n* = 3 mice per group. (C) Percentage of GAL‐PLGA (26 µm) and non‐galactosylated (22 µm) PLGA particle biodistribution in various organs. (D) Small particles (2 µm, white arrows) localize with F4/80‐positive cells (green) in liver tissue suggesting phagocytic uptake. The insert represents an expanded image. Scale bars 60 µm

Non‐galactosylated PLGA particles (mean diameter ~22 µm) were found exclusively in the lung (Figure 3A). In contrast, the galactosylated PLGA (GAL‐PLGA) targeted FGF7 delivery to the liver; 26‐µm diameter particles conferred exclusive hepatic localization. Smaller GAL‐PLGA particles (~2‐ and 10‐µm diameters) showed hepatic retention but not exclusively (Figure 3A–C). The smallest GAL‐PLGA particles (diameter ~2 µm) were engulfed by F4/80‐positive liver resident macrophages (Figure 3D). In parallel, the biodistribution of PLGA‐particles after injection into a tail vein was determined. A large proportion of the GAL‐PLGA‐particles (10‐µm diameter) were retained in the lung. Therefore, larger sizes (26‐µm diameter) of GAL‐PLGA particles were not tested for hepatic localization as it was reasoned that these two would be trapped in the lung (Figure S2A–C).

### 3.6 A FGF7‐GAL‐PLGA particle dose of 0.1 mg injected via the HPV was associated with a stable body weight and normal liver function tests

**FIGURE 4:**
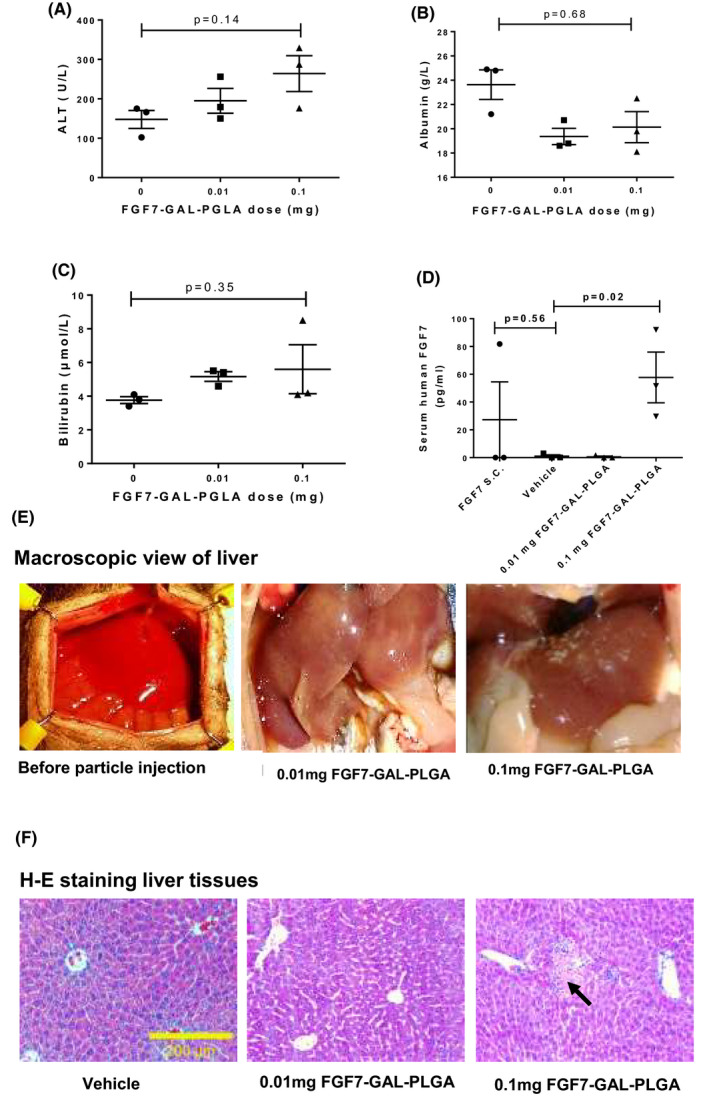
Dose response study shows that particles do not cause overt hepatotoxicity. (A) Serum ALT activity, (B) albumin, (C) bilirubin, and (D) human FGF7 levels from mice transplanted via HPV receiving FGF7‐GAL‐PLGA particles (26 μm; 0.01 and 0.1 mg) versus vehicle (30%, 100 μl FCS); additional groups in D mice receiving FGF7 s.c. 1.25 mg/kg s.c. × 2 doses. (E) Macroscopic view of the liver pre‐ and posttransplant with FGF7‐GAL‐PLGA particles. (F) H‐E staining liver (the black arrow indicates a necrotic patch). Mice were culled 72 h posttransplant. Data are mean ± SEM, *n* = 3 mice per group. HPV, hepatic portal vein

Mice receiving 0.01‐ and 0.1‐mg FGF7‐GAL‐PLGA particles via the HPV remained well with no demonstrable weight loss and no difference in serum albumin (marker of hepatocyte function), ALT, or bilirubin (markers of liver injury) at 72 h after transplant versus vehicle (Figure 4A–C), an effect that was still apparent 6 weeks posttransplant (Figure S3). Human FGF7 serum levels were detected at 24 h in mice transplanted with 0.1‐mg FGF7‐GAL‐PLGA particles (Figure 4D). In the group administered 0.1‐mg FGF7‐GAL‐PLGA particles, blood vessels in the liver appeared macroscopically milky white 24 h after injection via the HPV, and H&E staining exhibited occasional small necrotic areas (Figure 4E,F).

Higher doses of FGF7‐GAL‐PLGA particles (1 and 5 mg) were not associated with significant weight loss 24 h postinjection (Figure S4A), but an increase in liver injury markers, including ALT, bilirubin, and albumin, was observed (Figure S4B–D) with patchy liver necrosis (Figure S4E,F).

### 3.7 FGF7‐GAL‐PLGA particles (0.1 mg) significantly increased proliferation of liver cells at 72 h posttransplant with no increased proliferation at 6 weeks posttransplant

**FIGURE 5:**
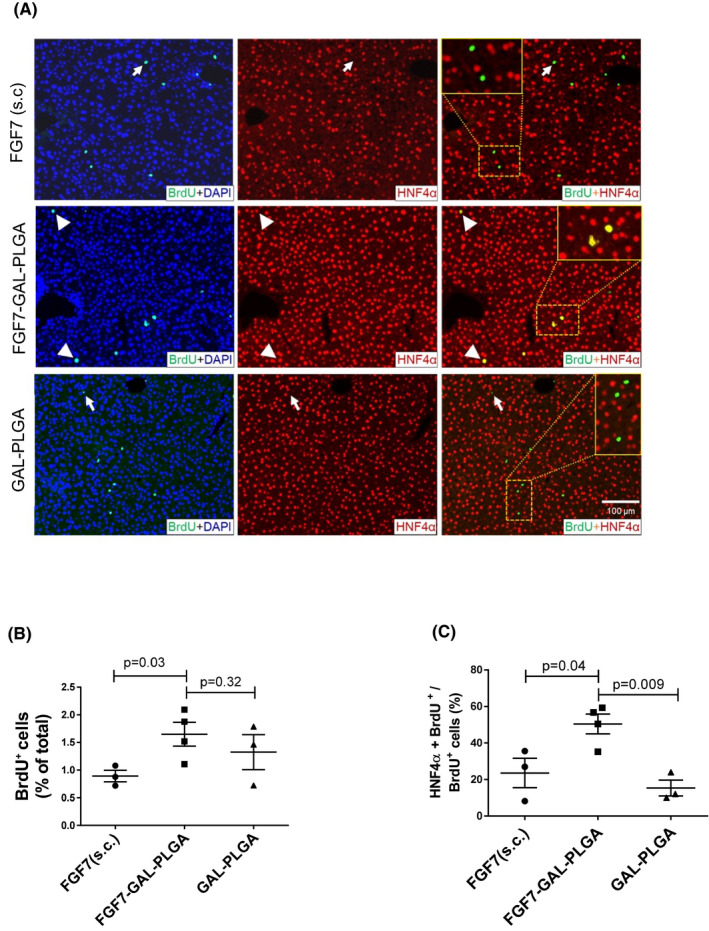
Effects of targeted FGF7‐GAL‐PLGA delivery via HPV versus FGF7 s.c. on liver cell proliferation. (A) FGF7 1.25 mg/kg s.c. ×2 doses, 0.1‐mg FGF7‐GAL‐PLGA (HPV) or GALPLGA alone (HPV), 30% FCS (HPV), 72 h following first injection. BrdU was administered 1 mg i.p. before cull. Representative micrographs of dual immunostaining applied on liver sections for BrdU (green, cell proliferation), HNF4α (red, hepatocytes), and DAPI (blue, nucleus staining). In the upper panels, white arrows show BrdU^+^ non‐parenchymal cells (HNF4α^−^), as magnified in the inset. In the middle row, mainly dual‐positive nuclei are observed (orange‐yellow), while in the lower panel (PLGA alone treated mice), the proliferating cells were mainly non‐parenchymal. Inset (×400) shows higher magnified regions of liver sections of different treatments. (B) Percentage of the proliferating (BrdU^+^) cells in each mouse group. (C) Fraction of proliferating hepatocytes (BrdU^+^, HNF4α^+^) to the total proliferating cells (BrdU^+^). Cell counting by Operetta system and Columbus. Scale 100 μm. *p < .05 using one‐way ANOVA Tukey's post hoc test

Based on the dose response studies, 0.1‐mg PLGA‐GAL‐FGF7 particles were administered via the HPV in mice and liver cell proliferation (total and hepatocyte) examined at 72 h. Co‐localization of BrdU^+^ and HNF4α^+^ cells in liver sections of mice that received FGF7‐GAL‐PLGA particles (Figure 5A) was 1.5‐fold greater than in mice receiving FGF7 1.25 mg/kg s.c. × 1 dose per day for 2 days. The greatest cell proliferation overall was observed in the liver of mice treated with FGF7‐GAL‐PLGA particles (Figure 5B, proportion [%] of proliferating cells in the liver [1.7 ± 0.1% vs. 0.9 ± 0.1%] in mice received FGF7 s.c.; *p* = .03) and 55% of proliferating cells were hepatocytes (*p* = .04, Figure 5C).

At 6 weeks, the % proliferating cells in the liver of mice treated with FGF7‐GAL‐PLGA particles was not significantly different versus control hyperglycemic mice (0.009 ± 0.002% vs. 0.015 ± 0.001%; *n* = 4, *p* = .13).

### 3.8 FGF7‐GAL‐PLGA particles (0.1 mg) transplanted with islets promoted early islet engraftment with improved long‐term glycemic control with no evidence of liver fibrosis

**FIGURE 6:**
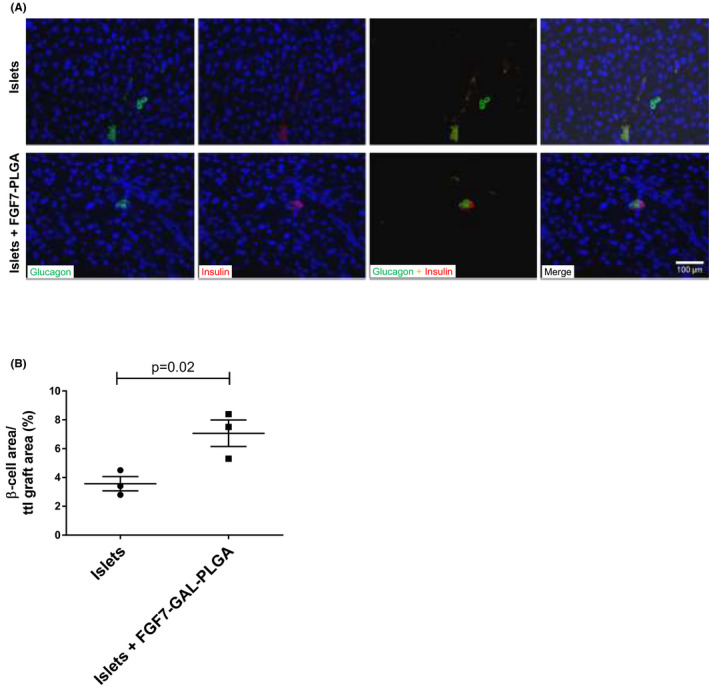
Islet detection in liver tissue 72 h post islet transplantation. Intraportal islet transplants, islets alone (*n* = 400), and islets (*n* = 400) co‐transplanted with FGF7‐GAL‐PLGA particles (0.1 mg) were performed and mice (*n* = 3 per group) culled 72 h posttransplant. (A) Dual immunofluorescence staining for islets (insulin‐β cells and glucagon‐α cells) in liver tissues. Scale 100 μm (Note: islets are exposed to sheer stress during transplantation accounting for fragmented appearance). (B) Average area of β‐cells from ≥8 FFPE sections from all four lobes of the liver were quantified from ≥15 non‐overlapping fields and expressed in terms of graft area (%). Each data point represents the average β‐cell to total graft area from 120 fields under 20× magnification. Mean ± SEM is shown. *p*‐value assessed by unpaired *t* test

**FIGURE 7:**
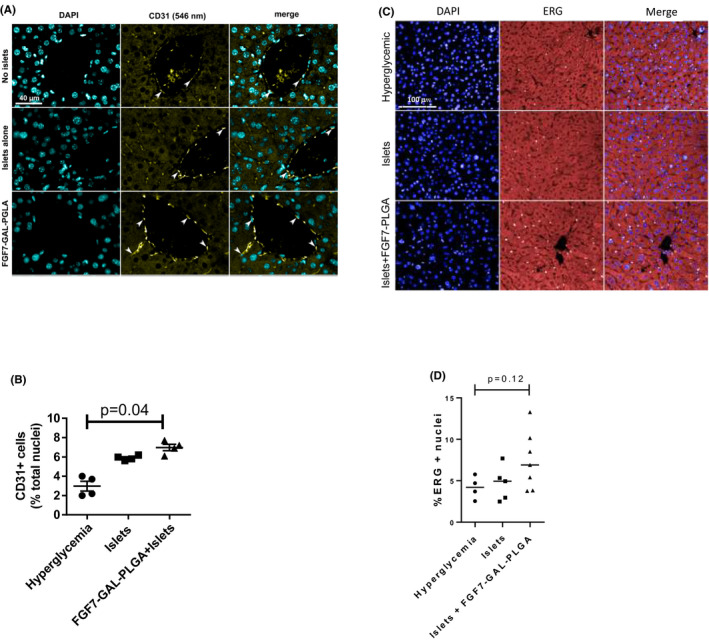
CD31 detection at 72 h and ERG detection at 6 weeks posttransplant in whole liver sections. At 72 h, posttransplant groups of mice were studied (*n* = 4/gp): (1) vehicle–hyperglycemic, (2) intraportal islet transplants alone (*n* = 400); (3) islets (*n* = 400) co‐transplanted with FGF7‐GAL‐PLGA particles (26‐µm diameter, 0.1 mg). (A) Immunofluorescence staining for CD31 nuclei in liver tissues. (B) Quantification of CD31 nuclei in groups. *p* = .04; one‐way ANOVA. At 6 weeks posttransplant, three groups of mice were studied (*n* = 5–8/gp): hyperglycemic‐vehicle, intraportal islet transplants alone (*n* = 400), and islets (*n* = 400) co‐transplanted with FGF7‐GAL‐PLGA particles (26‐µm diameter, 0.1 mg). (A) Immunofluorescence staining for ERG nuclei in liver tissues. (B) Quantification of ERG nuclei in all groups. *p* = .12; one‐way ANOVA

Greater numbers of islets were seen in the livers of hyperglycemic mice transplanted intraportally with islets and FGF7‐GAL‐PLGA particles versus islets alone as evidenced by greater numbers of dual insulin–glucagon‐positive cells in the liver 72 h posttransplant (Figure 6A,B, *p* = .02). At 72 h posttransplant, the percentage area of liver that was CD31‐positive was greatest in mice treated with FGF7‐GAL‐PLGA particles (0.1 mg) plus islets versus islets alone and versus GAL‐PLGA particles alone: 6.8 ± 0.9% versus 5.6 ± 0.6% versus 2.2 ± 0.06% (*p* = .04; Figure 7A,B). At 6 weeks, ERG and CD31^+^ staining was non‐significantly greatest in the livers of mice co‐transplanted with FGF7 particles with islets (Figure 7C,D, *p* = .12). There was no VEGF‐A staining in the livers quantifiable over the background compared to the isotype control in mice at 72 h and 6 weeks posttransplant (Figure S5A,B). VEGF‐A concentrations were detectable in serum and liver homogenates at 72 h and 6 weeks posttransplant with non‐significantly greater concentrations in mice transplanted with 5‐mg FGF7 particles (Figure S5C,D).

**FIGURE 8:**
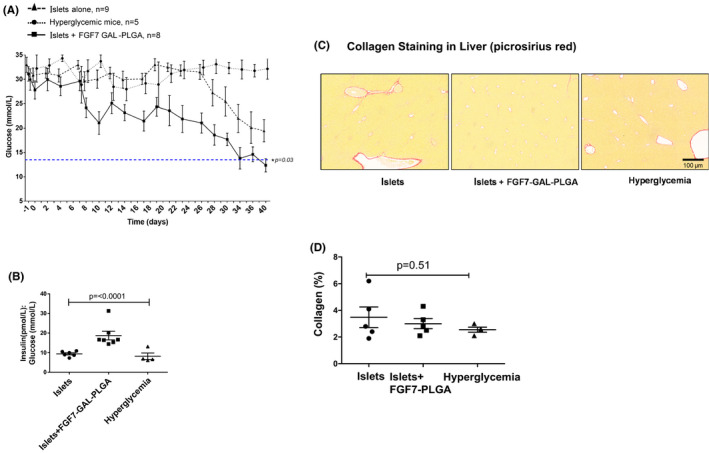
Biochemical assays and assessment of liver fibrosis in diabetic C57Bl/6 mice transplanted with a marginal islet mass ± FGF7‐GAL‐PLGA particles (0.1 mg) monitored for 6 weeks. (A) Non‐curative mass intraportal islet transplantation ± FGF7‐GAL‐PLGA particles (0.1 mg). Daily blood glucose concentrations are shown. Islets alone versus islets + FGF7‐GAL‐PLGA (0.1 mg) particles *p = .03 one‐way ANOVA. (B) Insulin:glucose ratio was significantly increased in the islet + FGF7‐GAL‐PLGA group. (C) Picrosirius red (PSR)‐stained liver tissues for fibrosis, (D) Percentage of collagen (red pixels) to liver tissue (yellow). Each value represents the mean of 8 (×10) fields per mouse liver section. Data were generated using an automated slide Scanner with InForm software

Mice receiving islets and FGF7‐GAL‐PLGA particles had tighter glycemic control versus those receiving islets alone with blood glucose levels normalizing by day 30 posttransplant (Figure 8A, *p* = .03) and with a greater proportion achieving a cure from their diabetes (75% vs. 0%; *p* < .001). Stimulated insulin concentrations at 60 min post i.p. GTT were not significantly different between the islet alone versus islet+FGF7‐GAL‐PLGA groups: median (IQR): 150 (143–170) vs. 155 (148–182) pmol/L, *p* = .37. When glucose concentrations were expressed in relation to insulin concentrations at 60 min post 2 g/kg i.p. GTT, however, mice co‐transplanted with FGF7 had greater insulin:glucose ratios (Figure 8B).

There was no difference in collagen content in the liver tissues between the groups at day 42 (Figure 8C,D). Serum biomarkers for liver injury at 6 weeks posttransplant were not different to controls (Figure S4A–C).

### 3.9 No evidence of pancreas regeneration 6 weeks posttransplant of islets ±galactosylated FGF7‐GAL‐PLGA particles (0.1 mg)

No proliferating ß‐cells were detected in the pancreases of mice treated with FGF7‐GAL‐PLGA particles (Figure S6A). There was no significant difference in pancreatic insulin content between groups (Figure S6B).

## 4 DISCUSSION

Transplantation of islets into patients with T1D stabilizes glycemic control, reducing SH^1^, ^5^, ^34^ but due to poor engraftment of islets into the liver^35^, ^36^ islets from two to three donor pancreases, a scarce resource, are required.

In diabetic rodents, partial hepatectomy preceding intra‐portal islet transplantation is associated with improved glycemic outcomes versus islet transplant alone, likely due to GF release, remodeling of the liver niche and liver cell proliferation, improving islet engraftment, and revascularization.^20^, ^30^, ^37^ However, partial hepatectomy is not a clinically applicable adjuvant therapy for intraportal islet transplantation in man. FGF7 RNA is expressed in most organs throughout the human body, with moderate expression in the pancreas and no expression in the islet in adulthood.^38^ FGFR2 is the cognate receptor for FGF7 and has been localized to the β cells of the islets of Langerhans^39^ but is absent from the alpha cells and the exocrine pancreas.^40^ Islets are mainly derived from the cells of the bud epithelium and FGF7 treatment activates ductal cell proliferation and their subsequent differentiation into β‐cells in human fetal pancreatic cell preparations.^41^ In an adult islet, however, exogenous FGF7 causes the ductal epithelium to proliferate but there is no evidence of endocrine differentiation or β‐cell proliferation.^42^ In our experiment with adult mouse donor islets, it is unlikely that adult intra‐islet ductal cells differentiated into β‐cells.

In our studies, FGF7 promoted proliferation of cells within the liver and it seems likely that this is one of the dominant mechanism promoting islet engraftment within the liver.

Of note, the doses of GFs used were based on safety and efficacy from our own experiments and the published literature and were not administered in equimolar amounts. The combination of FGF7, HGF, and T3 was less efficacious with respect to proliferation of liver cells versus FGF7 alone. We hypothesize that there is a balance between metabolic demand of the tissue and proliferation of cells in the liver with all three GF metabolic demand may be greater than with FGF7 alone impacting negatively on proliferation of liver cells.

Our results demonstrate increased vascularization of islets in the liver, with increased endothelial marker staining (CD31) in the liver at an early stage, coupled with improved glycemic control when the liver is targeted by FGF7 contained in galactosylated PLGA particles: the numbers of islets in the liver were greater in the mice receiving a portal injection of 0.1‐mg PLGA‐GAL‐FGF7 particles of 26‐µm diameter plus islets versus those receiving islets alone. Importantly, the dose of FGF7 received was approximately 60 ng over a 4‐week period representing approximately a 1000‐fold lower dose than the dose of 1.25 mg/kg FGF7 (×2 doses) administered subcutaneously to mice.

Furthermore, the majority of mice administered these particles were cured following islet transplantation. Importantly, there was no β‐cell regeneration in the native pancreas and FGF7 did not augment insulin secretion in our in vitro experiments, consistent with the beneficial effects being mediated by improved hepatic islet engraftment. Of note, there was no evidence of diminished OCRs from islets exposed to FGF7 and therefore no evidence of an adverse effect of FGF7 on islet function in the short term. Subcutaneous FGF7 increased hepatic non‐parenchymal cell proliferation; however, glycemic control was not improved following islet transplantation. FGF7 was not detectable systemically 24 h following subcutaneous administration and these short‐term effects may not be sufficient to increase islet engraftment. Islet engraftment, where blood vessels form between the islets and the liver, occurs largely between days 3 and 28.^43^ Subcutaneous FGF7 also causes cell proliferation in other organs including the lungs, pancreas, kidney, heart, and spleen, which limits clinical applicability.

When GAL‐PLGA particles were studied over a 21‐day period, the route of administration, particle size, and galactosylation influenced its sequestration within organs. The 26‐µm GAL‐PLGA particles delivered via the HPV route were sequestered solely in the liver. Smaller galactosylated particles (2 and 10 µm) delivered via the HPV route were sequestered in the liver and the capillary beds of the lungs, spleen, and other organs. Control PLGA particles of 22 µm without the galactosylated moiety were sequestered in the lung only, demonstrating that galactosylation is required for localization of the particle in the liver via ASGPR‐mediated endocytosis. When the 10‐µm GAL‐PLGA particles were delivered peripherally, a large proportion were trapped in the lung with only some reaching the liver; hence, this would not be a feasible way to translate this therapy into man.

In our short‐term experiments, FGF7 60 ng packaged into 0.1‐mg PLGA particles caused liver proliferation with no change in liver serum markers, although a minute patch of necrosis was seen which requires further exploration. With this dose of FGF7, 55% of proliferating cells were hepatocytes, contrasting with 22% when two doses of 1.25 mg/kg FGF7 were administered subcutaneously. Liver injury including patchy necrosis of the liver was demonstrable with 1‐ and 5‐mg FGF7‐GAL‐PLGA particles, suggesting that dose response studies in larger animal models may be useful before clinical studies are undertaken. Importantly, in the studies at 6 weeks, there was no evidence of hepatocyte proliferation, suggesting that modulation of the liver niche in the short‐term is sufficient for islet engraftment with no long‐term deleterious effects in the liver.

Numbers of islets transplanted^44^ along with younger donor age^45^ impact transplant outcome. This study demonstrates that modulation of the liver niche by FGF7‐GAL‐PLGA particles is a potential therapeutic strategy for increasing islet engraftment and islet transplant outcomes in man. FGF7 may increase engraftment of islets by stimulating angiogenesis via VEGF induction directly^21^ or indirectly^46^, ^47^ in keeping with increased hepatic CD31 staining at 72 h posttransplant in recipient mice. We did not detect VEGF‐A in liver; the immunofluorescence technique may not be sensitive enough to detect VEGF‐A at low levels or it may be upregulated at a different time point.

In this study, 0.1 mg of FGF7‐GAL‐PLGA particles would release ~40 ng of FGF7 in a mouse over 21 days leading to increased islet engraftment in the liver. FGF7 has FDA approval and held a license for severe oral mucositis in patients with hematologic malignancies receiving myelotoxic therapy. The recommended dose is ~25 mg intravenously for a 70 kg person over a 6‐day period.^48^ Extrapolating the dose of FGF7 administered in a mouse via particles direct to the liver to humans on a weight for weight basis, the dose used in man via the HPV would be >250‐fold lower than the licensed dose for treating oral mucositis. We believe this treatment can potentially be translated into man. Such a strategy would mean that islets isolated from just one donor pancreas may be sufficient to diminish hypoglycemia and stabilize glycemic control in patients with T1D, enabling more patients to be transplanted.

## DISCLOSURE

The authors of this manuscript have no conflicts of interest to disclose as described by the *American Journal of Transplantation*.

## AUTHOR CONTRIBUTIONS

SF, KS, and SJF conceptualized study and obtained funding for the study. OQ formulated FGF7 particles and drafted FGF7 particle methods; SA, PSL, AB, and JN performed animal experiments and laboratory assays. PSL performed immunohistochemistry for vascularization markers and performed additional statistical analyses. SA helped draft the manuscript and performed statistical analyses; JM, PB, and SFG gave technical laboratory assistance. SA, NM, and RC performed and analyzed oxygen consumption rate assays. SJF was PI for the liver regeneration studies, KMS was PI for the FGF7 particle formulation studies, and SF was PI for the FGF7 and metabolic studies; SF drafted and revised the manuscript and figures and performed statistical analyses for the in vivo transplant studies. All authors critically reviewed the manuscript. SF is the guarantor of this work and as such had full access to all the data in this study and takes responsibility for the integrity of the data and the accuracy of the data analysis.

## Supporting information

Supplementary Material

## Data Availability

Data available in article supplementary material.

## References

[1] Forbes S , McGowan NWA , Duncan K , et al. Islet transplantation from a nationally funded UK centre reaches socially deprived groups and improves metabolic outcomes. Diabetologia. 2015;58(6):1300‐1308. 25810037 10.1007/s00125-015-3554-3 PMC4415991

[2] Qi M , Kinzer K , Danielson KK , et al. Five‐year follow‐up of patients with type 1 diabetes transplanted with allogeneic islets: the UIC experience. Acta Diabetol. 2014;51(5):833‐843. 25034311 10.1007/s00592-014-0627-6 PMC4801517

[3] Shapiro AMJ , Lakey JRT , Ryan EA , et al. Islet transplantation in seven patients with type 1 diabetes mellitus using a glucocorticoid‐free immunosuppressive regimen. N Engl J Med. 2000;343(4):230‐238. 10911004 10.1056/NEJM200007273430401

[4] Forbes S , Oram RA , Smith A , et al. Validation of the BETA‐2 score: an improved tool to estimate beta cell function after clinical islet transplantation using a single fasting blood sample. Am J Transplant. 2016;16(9):2704‐2713. 27017888 10.1111/ajt.13807 PMC5074289

[5] Lablanche S , Vantyghem M‐C , Kessler L , et al. Islet transplantation versus insulin therapy in patients with type 1 diabetes with severe hypoglycaemia or poorly controlled glycaemia after kidney transplantation (TRIMECO): a multicentre, randomised controlled trial. Lancet Diabetes Endocrinol. 2018;6(7):527‐537. 29776895 10.1016/S2213-8587(18)30078-0

[6] Forbes S , Senior PA , Shapiro AMJ . Islet transplantation in type 1 diabetes: moving forward. Lancet Diabetes Endocrinol. 2018;6(7):516‐517. 29776896 10.1016/S2213-8587(18)30107-4

[7] Hering BJ , Clarke WR , Bridges ND , et al. Phase 3 trial of transplantation of human islets in type 1 diabetes complicated by severe hypoglycemia. Diabetes Care. 2016;39(7):1230‐1240. 27208344 10.2337/dc15-1988 PMC5317236

[8] Brennan DC , Kopetskie HA , Sayre PH , et al. Long‐term follow‐up of the edmonton protocol of islet transplantation in the United States. Am J Transplant. 2016;16(2):509‐517. 26433206 10.1111/ajt.13458

[9] Barshes NR , Wyllie S , Goss JA . Inflammation‐mediated dysfunction and apoptosis in pancreatic islet transplantation: implications for intrahepatic grafts. J Leukoc Biol. 2005;77(5):587‐597. 15728243 10.1189/jlb.1104649

[10] Forbes S , Bond AR , Thirlwell KL , et al. Human umbilical cord perivascular cells improve human pancreatic islet transplant function by increasing vascularization. Sci Transl Med. 2020;12(526):eaan5907. 31941825 10.1126/scitranslmed.aan5907

[11] Rother KI , Harlan DM . Challenges facing islet transplantation for the treatment of type 1 diabetes mellitus. J Clin Invest. 2004;114(7):877‐883. 15467822 10.1172/JCI23235 PMC518676

[12] Biarnes M , Montolio M , Nacher V , Raurell M , Soler J , Montanya E . Beta‐cell death and mass in syngeneically transplanted islets exposed to short‐ and long‐term hyperglycemia. Diabetes. 2002;51(1):66‐72. 11756324 10.2337/diabetes.51.1.66

[13] Kanak MA , Takita M , Kunnathodi F , Lawrence MC , Levy MF , Naziruddin B . Inflammatory response in islet transplantation. Int J Endocrinol. 2014;2014:451035. 24883060 10.1155/2014/451035 PMC4021753

[14] Hammond JS , Gilbert TW , Howard D , et al. Scaffolds containing growth factors and extracellular matrix induce hepatocyte proliferation and cell migration in normal and regenerating rat liver. J Hepatol. 2011;54(2):279‐287. 21126791 10.1016/j.jhep.2010.06.040

[15] Shimoda M , Chen S , Noguchi H , Matsumoto S , Grayburn PA . In vivo non‐viral gene delivery of human vascular endothelial growth factor improves revascularisation and restoration of euglycaemia after human islet transplantation into mouse liver. Diabetologia. 2010;53(8):1669‐1679. 20405100 10.1007/s00125-010-1745-5 PMC3804430

[16] Forbes SJ , Themis M , Alison MR , Selden C , Coutelle C , Hodgson HJ . Retroviral gene transfer to the liver in vivo during tri‐iodothyronine induced hyperplasia. Gene Ther. 1998;5(4):552‐555. 9614581 10.1038/sj.gt.3300613

[17] Alwahsh SM , Rashidi H , Hay DC . Liver cell therapy: is this the end of the beginning? Cell Mol Life Sci. 2018;75(8):1307‐1324. 29181772 10.1007/s00018-017-2713-8 PMC5852182

[18] Forbes SJ , Themis M , Alison MR , Sarosi I , Coutelle C , Hodgson HJ . Synergistic growth factors enhance rat liver proliferation and enable retroviral gene transfer via a peripheral vein. Gastroenterology. 2000;118(3):591‐598. 10702211 10.1016/s0016-5085(00)70266-6

[19] Takase HM , Itoh T , Ino S , et al. FGF7 is a functional niche signal required for stimulation of adult liver progenitor cells that support liver regeneration. Genes Dev. 2013;27(2):169‐181. 23322300 10.1101/gad.204776.112 PMC3566310

[20] Saito Y , Chan NK , Hathout E . Partial hepatectomy improves the outcome of intraportal islet transplantation by promoting revascularization. Islets. 2012;4(2):138‐144. 22622159 10.4161/isl.19491 PMC3396702

[21] Xiong Y , Scerbo MJ , Seelig A , et al. Islet vascularization is regulated by primary endothelial cilia via VEGF‐A‐dependent signaling. eLife. 2020;9:e56914. 33200981 10.7554/eLife.56914 PMC7695455

[22] Liu S , Zhang L , Cheng J , Lu Y , Liu J . Sustained release of hepatocyte growth factor by cationic self‐assembling peptide/heparin hybrid hydrogel improves beta‐cell survival and function through modulating inflammatory response. Int J Nanomedicine. 2016;11:4875‐4890. 27729786 10.2147/IJN.S108921 PMC5042198

[23] Fiaschi‐Taesch N , Stewart AF , Garcia‐Ocana A . Improving islet transplantation by gene delivery of hepatocyte growth factor (HGF) and its downstream target, protein kinase B (PKB)/Akt. Cell Biochem Biophys. 2007;48(2–3):191‐199. 17709889 10.1007/s12013-007-0024-7

[24] Panoskaltsis‐Mortari A , Taylor PA , Rubin JS , et al. Keratinocyte growth factor facilitates alloengraftment and ameliorates graft‐versus‐host disease in mice by a mechanism independent of repair of conditioning‐induced tissue injury. Blood. 2000;96(13):4350‐4356. 11110712

[25] Huang Z , Zhu G , Sun C , et al. A novel solid‐phase site‐specific PEGylation enhances the in vitro and in vivo biostability of recombinant human keratinocyte growth factor 1. PLoS One. 2012;7(5):e36423. 22574160 10.1371/journal.pone.0036423 PMC3344868

[26] Fredenberg S , Wahlgren M , Reslow M , Axelsson A . The mechanisms of drug release in poly(lactic‐co‐glycolic acid)‐based drug delivery systems–a review. Int J Pharm. 2011;415(1–2):34‐52. 21640806 10.1016/j.ijpharm.2011.05.049

[27] Ashwell G , Morell AG . The role of surface carbohydrates in the hepatic recognition and transport of circulating glycoproteins. Adv Enzymol Relat Areas Mol Biol. 1974;41:99‐128. 4609051 10.1002/9780470122860.ch3

[28] Li Y , Huang G , Diakur J , Wiebe LI . Targeted delivery of macromolecular drugs: asialoglycoprotein receptor (ASGPR) expression by selected hepatoma cell lines used in antiviral drug development. Curr Drug Deliv. 2008;5(4):299‐302. 18855599 10.2174/156720108785915069

[29] Jackson Labs . Insulin content by acid‐ethanol extraction. Animal models of diabetic complications consortium protocols. https://www.diacomp.org/shared/document.aspx?id=73&docType=Protocol. Published 2009. Accessed January 21, 2021.

[30] Sudo T , Hiyama E , Murakami Y , Yokoyama Y , Takesue Y , Sueda T . Hepatic regeneration promotes engraftment of intraportally transplanted islet cells. Surgery. 2005;137(6):612‐619. 15933627 10.1016/j.surg.2005.02.007

[31] Raven A , Lu WY , Man TY , et al. Cholangiocytes act as facultative liver stem cells during impaired hepatocyte regeneration. Nature. 2017;547(7663):350‐354. 28700576 10.1038/nature23015 PMC5522613

[32] Pusztaszeri MP , Seelentag W , Bosman FT . Immunohistochemical expression of endothelial markers CD31, CD34, von Willebrand factor, and Fli‐1 in normal human tissues. J Histochem Cytochem. 2006;54(4):385‐395. 16234507 10.1369/jhc.4A6514.2005

[33] Haber MA , Iranmahboob A , Thomas C , Liu M , Najjar A , Zagzag D . ERG is a novel and reliable marker for endothelial cells in central nervous system tumors. Clin Neuropathol. 2015;34(3):117‐127. 25881913 10.5414/NP300817 PMC4542182

[34] Brooks AM , Walker N , Aldibbiat A , et al. Attainment of metabolic goals in the integrated UK islet transplant program with locally isolated and transported preparations. Am J Transplant. 2013;13(12):3236‐3243. 24119216 10.1111/ajt.12469

[35] Gala‐Lopez BL , Neiman D , Kin T , et al. Beta cell death by cell‐free DNA and outcome after clinical islet transplantation. Transplantation. 2018;102(6):978‐985. 29329189 10.1097/TP.0000000000002083

[36] Lehmann‐Werman R , Neiman D , Zemmour H , et al. Identification of tissue‐specific cell death using methylation patterns of circulating DNA. Proc Natl Acad Sci USA. 2016;113(13):E1826‐E1834. 26976580 10.1073/pnas.1519286113 PMC4822610

[37] Michalopoulos GK . Liver regeneration after partial hepatectomy: critical analysis of mechanistic dilemmas. Am J Pathol. 2010;176(1):2‐13. 20019184 10.2353/ajpath.2010.090675 PMC2797862

[38] Proteinatlas.org . Protein atlas FGF7 and tissue. https://wwwproteinatlasorg/ENSG00000140285‐FGF7/tissue. Accessed December 18, 2020.

[39] Proteinatlas.org . Proteinatlas FGF2R in pancreas. https://wwwproteinatlasorg/ENSG00000066468‐FGFR2/tissue/pancreas. Accessed December 18, 2020.

[40] Hart AW , Baeza N , Apelqvist A , Edlund H . Attenuation of FGF signalling in mouse beta‐cells leads to diabetes. Nature. 2000;408(6814):864‐868. 11130726 10.1038/35048589

[41] Percival AC , Slack JM . Analysis of pancreatic development using a cell lineage label. Exp Cell Res. 1999;247(1):123‐132. 10047454 10.1006/excr.1998.4322

[42] Yi ES , Yin S , Harclerode DL , et al. Keratinocyte growth factor induces pancreatic ductal epithelial proliferation. Am J Pathol. 1994;145(1):80‐85. 7913296 PMC1887296

[43] Hathout E , Chan NK , Tan A , et al. In vivo imaging demonstrates a time‐line for new vessel formation in islet transplantation. Pediatr Transplant. 2009;13(7):892‐897. 19017287 10.1111/j.1399-3046.2008.01088.x PMC2837508

[44] Ryan EA , Paty BW , Senior PA , et al. Five‐year follow‐up after clinical islet transplantation. Diabetes. 2005;54(7):2060‐2069. 15983207 10.2337/diabetes.54.7.2060

[45] Niclauss N , Bosco D , Morel P , et al. Influence of donor age on islet isolation and transplantation outcome. Transplantation. 2011;91(3):360‐366. 21344706 10.1097/tp.0b013e31820385e6

[46] Beer HD , Gassmann MG , Munz B , et al. Expression and function of keratinocyte growth factor and activin in skin morphogenesis and cutaneous wound repair. J Invest Dermatol Symp Proc. 2000;5(1):34‐39. 10.1046/j.1087-0024.2000.00009.x 11147673

[47] Frank S , Hubner G , Breier G , Longaker MT , Greenhalgh DG , Werner S . Regulation of vascular endothelial growth factor expression in cultured keratinocytes. Implications for normal and impaired wound healing. J Biol Chem. 1995;270(21):12607‐12613. 7759509 10.1074/jbc.270.21.12607

[48] Kepivance in oral Mucositis. https://reference.medscape.com/drug/kepivance‐palifermin‐342271 PR. Accessed December 18, 2020.

