# Supplementary material for: Fibroblast growth factor 7 releasing particles enhance islet engraftment and improve metabolic control following islet transplantation in mice with diabetes
Source: Am J Transplant. 2021 Feb 2;21(9):2950–63. doi: 10.1111/ajt.16488 (PMC8603932; doi:10.1111/ajt.16488)
Supplement: Supplementary file 1 — Supplementary Material [file AJT-21-2950-s001.docx]

**Change in weight and glucose in C57Bl/6 mice receiving FGF7 5mg/kg**

**Suppl. Table 1**

| Mouse weight  pre-injection (g) | % weight change | BG  pre-injection (mmol/l) | Lowest BG recorded post injection | Comments |
| --- | --- | --- | --- | --- |
| 29.4 | 5 | 9.3 | 8.4 | - |
| 31.4 | 22 | 7.7 | 3.2 | Culled day 4 anorectic and weight loss |
| 31.8 | 9 | 7.6 | 7.0 | - |
| 29.4 | 24 | 8.5 | 3.0 | Culled day 5 anorectic and weight loss |
| 33.7 | 11 | 8.9 | 8.2 | - |
| 31.0 | 21 | 12.2 | 3.5 | Culled day 9 anorectic and weight loss. |
| 30.0 | 7 | 9.2 | 8.3 | - |
| 31.5 | 5 | 9.4 | 8.9 | - |

**Suppl. Table 2**

Nomoglycaemic mice were given a single injection s.c. of FGF7 5mg/kg. The experiment was terminated at day 9 post injection. Decrease in weight and blood glucose was greater in the FGF7 injected mice vs. controls (vehicle injection) (both p<0.01).

## **Primary antibodies used in immunofluorescence staining**

| Reagent | Clone | Dilution | Source |
| --- | --- | --- | --- |
| Rat anti-BrdU | BU1/75 (ICR1) | 1:100 | Abcam |
| Mouse anti-insulin | K36AC10 | 1:800 | Sigma |
| Rabbit anti-Ki-67 | SP6 | 1:150 | Abcam |
| Mouse anti-HNF4α | H1415 | 1:200 | Perseus Proteomics |
| Rat anti-F4/80 | A3-1 | 1:50 | Abcam |
| Mouse anti-glucagon | K79bB10 | 1:1500 | Abcam |
| Rabbit anti-ERG (CD31) | EPR3864 | 1:100 | Abcam |
| VEGF-A | EP1176Y | 1:100 | Abcam |

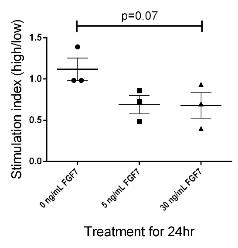


**A**

**Suppl. Fig.1 Insulin secretion rates and OCRs of islets following incubation with FGF7 at 5 and 30ng/ml versus controls for 24 hours.** n=20 islets per well for insulin secretion studies and n=30 islets per well for OCR studies were examined in triplicate in each of the groups. **(A)** Glucose Stimulation Index is shown – this represents the insulin secreted at high glucose divided by insulin secreted at low glucose. **(B)** OCR across a mitochondrial stress test, from islets exposed to 0, 5 and 30ng/ml FGF7. Data are mean± SEM, statistical analysis was performed by one way ANOVA-Tukey’s post hoc. There were no statistically significant differences between the groups.

**B**

p=0.29

**Suppl. Fig. 1**

**A**

**Suppl. Fig.2 - Intravenous (tail vein) injection of 10µm GAL-PLGA particles stay predominantly in the lung and spleen.** PLGA particles (non-galactosylated 2µm, 22µm and galactosylated 10µm) (1 mg, i.v.) and control vehicle (saline) were injected into mice to investigate particle distribution in tissues. **(A)** Representative fluorescent images (x40) of organs (indicated left) extracted 24 hour after injection of different bioparticle formulations. Cryosections (30 µm) were fixed and stained with DAPI (blue) to label cell nuclei. Fluorescent particles (red epifluorescence) are highlighted (white arrows). Galactosylated particles of 10 µm were trapped in the microcirculation of the lungs and spleen and therefore larger diameter particles were not tested. **(B)** Particles quantified per tissue grouped by formulation. Average particle counts (from 11 slides) quantified per tissue grouped in formulations indicated. **(C)** The percentage distribution of particles between tissues. Non-gal – non-galactosylated. GAL- Galactosylated.

**C**

**B**

**Suppl. Fig. 2**

**A**

**Suppl. Fig. 3. Serum biochemistry 6 weeks post-transplantation of islets ± 0. 1 mg FGF7-GAL-PLGA particles. (A)** ALT, **(B)** AST, **(C)** albumin. Data are mean± SEM, and statistical analysis was performed by one way ANOVA-Tukey’s post hoc. There were no statistically significant differences between the groups.


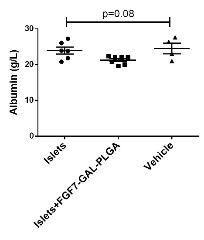

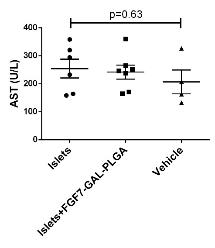

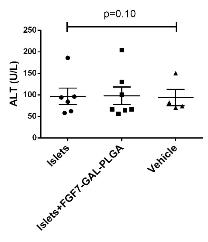


**A**

**B**

**C**

**Suppl. Fig. 3**

1mg FGF7-GAL-PLGA

5mg FGF7-GAL-PLGA

1mg FGF7-GAL-PLGA

5mg FGF7-GAL-PLGA

**F**

**Suppl. Fig 4. Administration of 1 mg and 5 mg FGF7-GAL-PLGA particles via the HPV.** Mice were transplanted with 1 mg and 5 mg FGF7-GAL-PLGA particles along with GAL-PLGA (0) alone via the HPV and culled 72hrs post-transplant. **(A)** Percentage weight loss of mice 24 hrs after particle administration. **(B)** Alanine aminotransferase, **(C)** bilirubin, and **(D)** albumin. **(E)** Macroscopic view of the liver at time of mouse cull for each treatment, and corresponding microscopic H-E staining for liver sections showing necrosis with high doses **(F)**. N=2-3 mice / group. Blue arrows - patchy areas. Data are mean± SEM, and statistical analysis was performed by one way ANOVA-Tukey’s post hoc. There were no statistically significant differences between the groups.

**Suppl. Fig. 4**

**E**


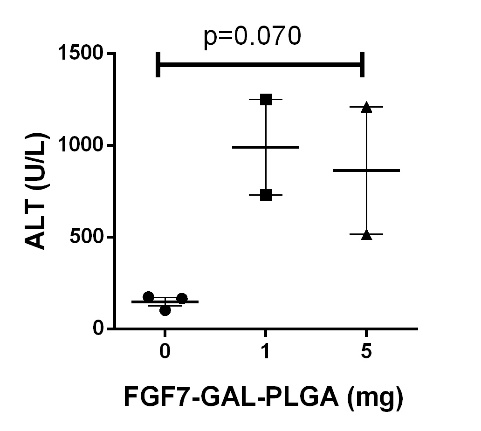

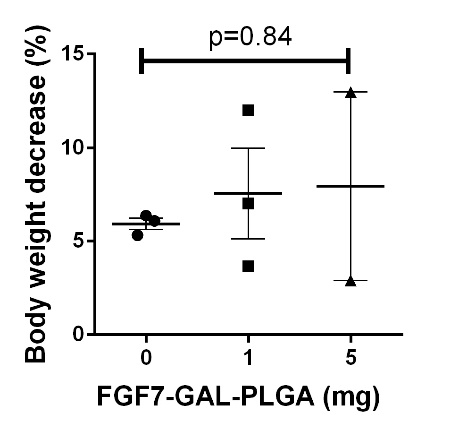


**B**

**D**

**C**

**A**


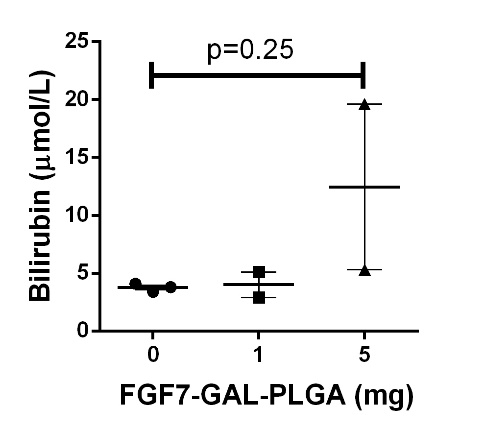

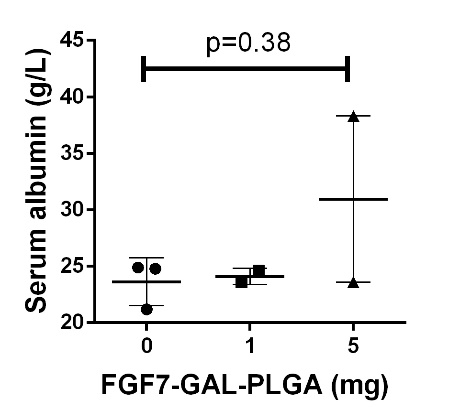


**Suppl. Fig 3. Administration of 1 mg and 5 mg FGF7-GAL-PLGA particles via the HPV.** Mice were transplanted with 1 mg and 5 mg FGF7-GAL-PLGA particles along with GAL-PLGA (0) alone via the HPV. **(A)** Percentage weight loss of mice 24 hrs after particle administration. **(B)** Alanine aminotransferase, **(C)** bilirubin, and **(D)** albumin. **(E)** Macroscopic view of the liver at time of mouse cull for each treatment, and corresponding microscopic H-E staining for liver sections **(F)**. N=2-3 mice / group. Blue arrows - patchy areas.

**A**

**B**

**Suppl. Fig. 5**

**C**

**D**

**Suppl. Fig. 5 (A) VEGF-A staining of liver (top), heart (right), spleen (bottom), lung (lower left).** Organs were stained for VEGF-A as described and imaged using the Operetta High-Content System. Strong autofluorescence was evident in heart and spleen. **(B) VEGF-A staining of liver:** Livers were step sectioned (5μm) from mice at 72hr and 6 weeks post-transplant and stained for VEGF-A; VEGF-A staining was not quantifiable over the background compared to the isotype control. The autofluorescence noted on image analysis was only mildly attenuated by switching to far-red secondary antibody (AlexaFluor 647). **(C) VEGF-A concentrations in serum:** VEGF-A concentrations were not significantly raised with FGF7-GAL-PLGA particles at 72hrs or 6 weeks post-transplant (data at 72hrs shown). **(D**) **VEGF-A concentrations in liver homogenates:** VEGF-A concentrations were not significantly raised in liver homogenates. Islets+FGF – islets+FGF-GAL-PLGA

**Suppl. Fig. 6**

**A**

**
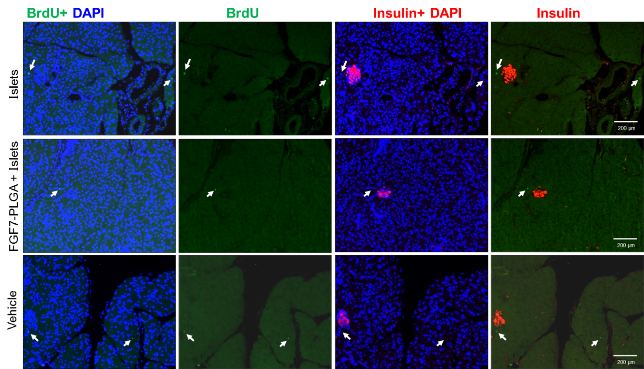
**

**B**

**Suppl. Fig. 6 - Regeneration and insulin content of pancreases from diabetic C57Bl/6 mice transplanted with a non-curative islet mass ± FGF7-GAL-PLGA particles (0.1mg) assessed at 6 weeks post transplant. (A)** Dual immunofluorescence staining for insulin and BrdU on pancreatic tissue. Representative micrographs show basal proliferation in all groups. White arrows - BrdU^+^ nuclei (proliferating cells) in the pancreas. No dual positive cells (Insulin^+^ BrdU^+^ / Ki-67^+^) were detected by Operetta imaging. **(B)** Pancreatic insulin levels normalised to protein content. Data represents mean ± SEM, n= 6 mice per group.

**Suppl. Fig. 6. No evidence of pancreatic regeneration at 6 weeks post-transplantation of islets ± galactosylated FGF7-GAL-PLGA particles (0.1mg). (A)** BrdU, Insulin and DAPI staining in mouse pancreases following treatment with FGF7-GAL-PLGA and islets (n=4), islets only (n=5), or vehicle. **(B)** No significant difference in pancreatic insulin content was observed between groups (n=4/gp). Data is mean ±SEM.
